# A novel macrophage-mediated biomimetic delivery system with NIR-triggered release for prostate cancer therapy

**DOI:** 10.1186/s12951-019-0513-z

**Published:** 2019-07-10

**Authors:** Lei Qiang, Zheng Cai, Wenjun Jiang, Jiyong Liu, Zongguang Tai, Guorui Li, Chunai Gong, Shen Gao, Yuan Gao

**Affiliations:** 1 Department of Pharmacy, Changhai Hospital, Second Military Medical University, Shanghai, 200433 China; 2 Department of Clinical Pharmacy and Pharmaceutical Management, School of Pharmacy, Fudan University, Shanghai, 201203 China

**Keywords:** Photothermal effect, Biomimetic delivery system (BDS), Macrophage, Cancer therapy

## Abstract

**Background:**

Macrophages with tumor-tropic migratory properties can serve as a cellular carrier to enhance the efficacy of anti neoplastic agents. However, limited drug loading (DL) and insufficient drug release at the tumor site remain the main obstacles in developing macrophage-based delivery systems. In this study, we constructed a biomimetic delivery system (BDS) by loading doxorubicin (DOX)-loaded reduced graphene oxide (rGO) into a mouse macrophage-like cell line (RAW264.7), hoping that the newly constructed BDS could perfectly combine the tumor-tropic ability of macrophages and the photothermal property of rGO.

**Results:**

At the same DOX concentration, the macrophages could absorb more DOX/PEG-BPEI-rGO than free DOX. The tumor-tropic capacity of RAW264.7 cells towards RM-1 mouse prostate cancer cells did not undergo significant change after drug loading in vitro and in vivo. PEG-BPEI-rGO encapsulated in the macrophages could effectively convert the absorbed near-infrared light into heat energy, causing rapid release of DOX. The BDS showed excellent anti-tumor efficacy in vivo.

**Conclusions:**

The BDS that we developed in this study had the following characteristic features: active targeting of tumor cells, stimuli-release triggered by near-infrared laser (NIR), and effective combination of chemotherapy and photothermotherapy. Using the photothermal effect produced by PEG-BPEI-rGO and DOX released from the macrophages upon NIR irradiation, MAs-DOX/PEG-BPEI-rGO exhibited a significant inhibitory effect on tumor growth.

## Background

In recent years, biomimetic delivery systems (BDS) using endogenous cells to transport therapeutic agents to the tumor sites have attracted much attention [1–3]. Solid tumors with high chemokine-secreting tissues can attract a large number of mesenchymal stem cells and immunocytes to the tumor site [4, 5]. Among the endogenous cells, macrophages have been reported as a tumor-targeted transport vector and attracted extensive research attention due to their strong phagocytic capacity and tolerance to chemotherapeutic drugs [6, 7]. Some studies have confirmed the feasibility of using macrophages to transport chemotherapeutic drugs or drug-loaded nanoparticles to the tumor site [8–11]. However, the low drug-loading capacity and insufficient drug release at the tumor site have limited the use of macrophages as a tumor targeting vector [12].

Reduced graphene oxide (rGO) has attracted extensive attention in recent years due to its special physiochemical properties [13]. Other than its high drug-loading capacity, rGO also has a strong photothermal conversion efficiency, which could efficiently convert the absorbed light energy into heat, thus causing the disruption of bond energy and release of the loaded drug [14]. rGO nanoparticles with high loading capacity for chemical drugs and excellent NIR photothermal conversion efficiency have been reported as an ideal platform for NIR-controlled drug delivery system [15, 16].

**Fig. 1:**
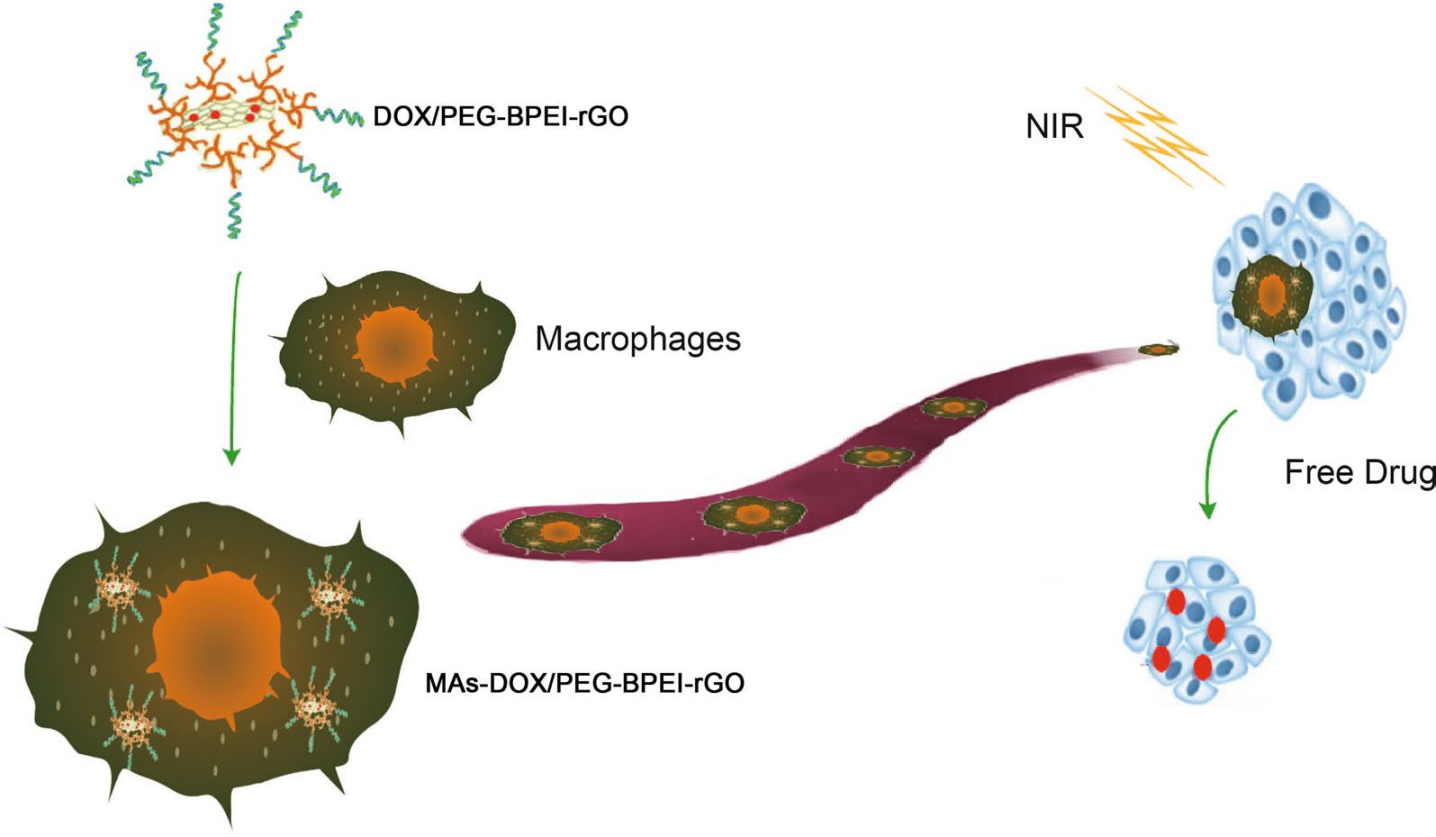
Schematic illustration of macrophages loading DOX/PEG-BPEI-rGO to target tumor site and initiate drug release after NIR irradiation

However, rGO is an exogenous substance that can be easily recognized and cleared by the reticuloendothelial system. In our study (Fig. 1), doxorubicin (DOX), a model anti-cancer drug was loaded onto rGO (DOX/rGO), followed by internalization of macrophages to obtain a macrophage-based biomimetic drug delivery system (MAs-DOX/rGO). This strategy combines the natural function of macrophages and the property of rGO. DOX/rGO is transported to the tumor site by means of chemotaxis of the macrophages. Upon NIR irradiation, the dissociated DOX and the heat produced by rGO would destroy the macrophages. As a result, DOX would be excreted to the surrounding environment in the form of free drug. This novel biomimetic drug delivery system can simultaneously deliver two therapeutic factors (the chemotherapy drug and heat) to tumor cells in a NIR-controlled way [17]. RAW264.7 cells, a kind of mouse macrophage-like cell line, were used to construct the delivery system. The tumor targeting ability of the drug-loaded macrophages was investigated in vitro and in vivo, and anti-cancer efficacy research was conducted in a mouse prostate cancer model.

## Results

### Characterization of PEG-BPEI-rGO (rGO)

**Fig. 2:**
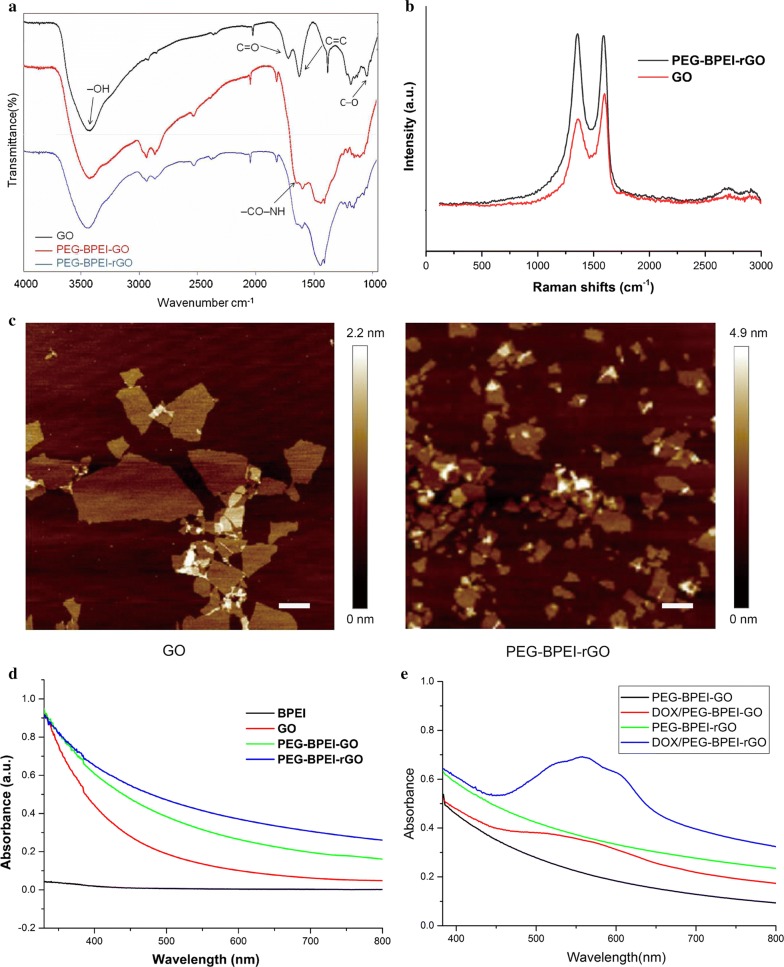
**a** FT-IR spectra of GO, PEG-BPEI-GO and PEG-BPEI-rGO. **b** Raman spectra of GO and PEG-BPEI-rGO. **c** AFM images of GO and PEG-BPEI-rGO, the scale bar is 1 μm. **d** UV–Vis spectra of BPEI, GO, PEG-BPEI-GO and PEG-BPEI-rGO. **e** UV/vis absorbance spectra of solutions of PEG-BPEI-rGO, DOX/PEG-BPEI-rGO, PEG-BPEI-GO, and DOX/PEG-BPEI-GO

The successful covalent bonding of BPEI to carboxylic group of GO and the reduction process were proved by FT-IR spectroscopy (Fig. 2a). The GO spectrum revealed the existence of –OH (3400 cm^−1^), C=O (1733 cm^−1^), C=C (1590 cm^−1^), and C–O (1050 cm^−1^). In the FT-IR spectrum of PEG-BPEI-GO, the appearance of the vibration (1650 cm^−1^) and the disappearance of carboxylic group bands (1733 cm^−1^) confirmed the formation of amide linkages [18]. After chemical reduction, the band at 1050 cm^−1^ due to the C–O stretching in GO was slightly diminished. To further confirm the reduction of GO, Raman spectroscopy was carried out. As shown in Fig. 2b, there are two representative peaks at 1370 cm^−1^ and 1620 cm^−1^, representing band D and band G. In general, the disorder degree of graphene sheets was indicated by the I_D_/I_G_ ratio. The I_D_/I_G_ ratio increased from 0.91 to 1.32 after chemical conjugation and reduction, indicating that more numerous sp^2^ domains were formed. The morphology and size of GO and PEG-BPEI-rGO were investigated by AFM (Fig. 2c). The size of GO before modification was about 300 nm ~ 2 μm. After modification and reduction, the size of GO decreased to about 100 ~ 600 nm, while the thickness increased. Size reduction and thickness increase may be due to the folding and reforming of GO during the connecting and reduction process.

Knowing that excellent optical absorbance is the basic condition of PEG-BPEI-rGO as photothermal material [19], we examined the UV/vis absorbance spectra of GO, PEG-BPEI-GO, and PEG-BPEI-rGO. As shown in Fig. 2d, all the GO-containing materials exhibited obvious absorption over a wide range. The absorption of PEG-BPEI-rGO increased over the whole detection range, compared with the unreduced form. These results may be due to the partially recovered conjugated aromatic clusters during the chemical reduction process. We investigated the ultraviolet spectra of PEG-BPEI-GO and PEG-BPEI-rGO after drug loading. As shown in Fig. 2e, the characteristic absorption of DOX is at 550 nm, and the absorption of DOX/PEG-BPEI-rGO at 550 nm is obviously enhanced after drug loading, indicating that DOX is successfully loaded onto the carrier. Compared with DOX/PEG-BPEI-GO, the absorption of DOX/PEG-BPEI-rGO at 550 nm was significantly enhanced, indicating that the drug loading capacity of PEG-BPEI-rGO was significantly increased. The DL capacity of PEG-BPEI-rGO was measured by UV spectrophotometry. Consistent with the literature reports [20], PEG-BPEI-rGO showed a high drug loading capacity about 62%.

**Fig. 3:**
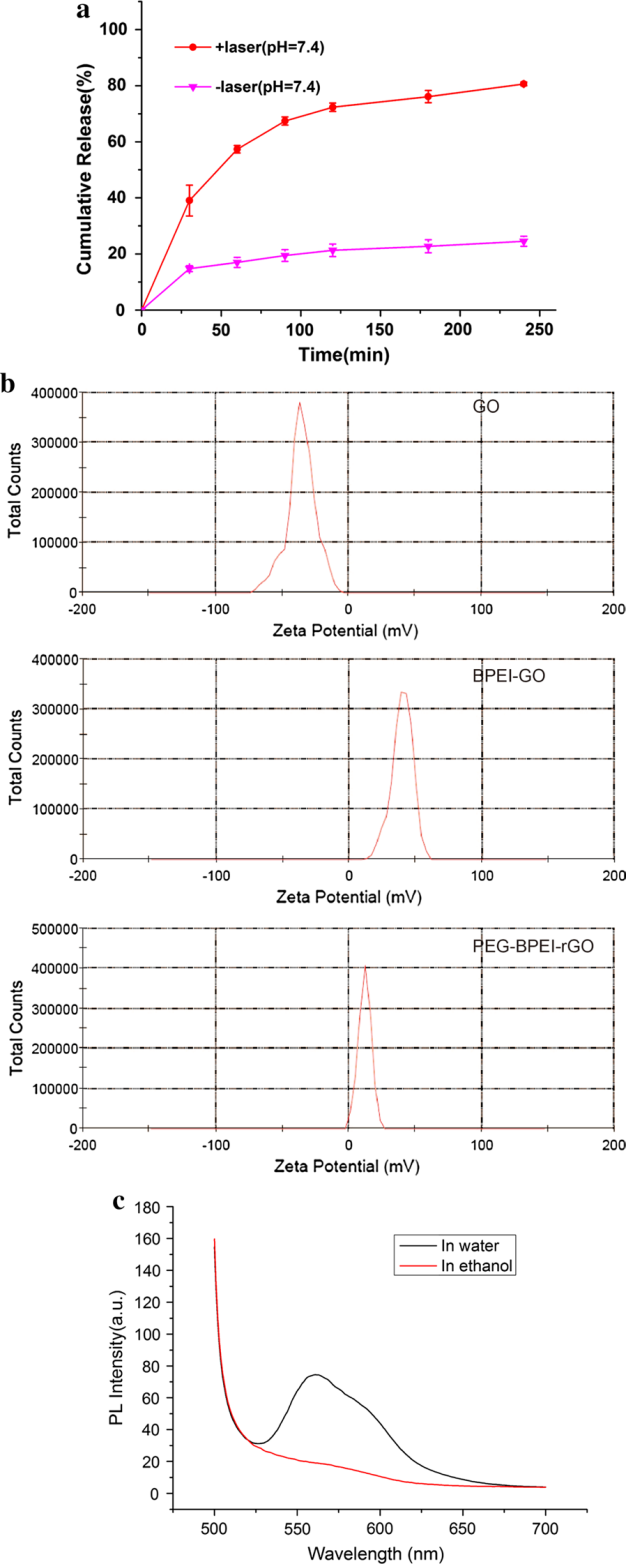
**a** Drug release curves of DOX from DOX/PEG-BPEI-rGO with or without NIR irradiation. **b** The zeta potential of GO, BPEI-GO and PEG-BPEI-rGO. **c** Fluorescence spectra of DOX after adsorption on PEG-BPEI-rGO in water and ethanol

We studied the in vitro drug release of DOX/PEG-BPEI-rGO. Under the condition of pH = 7.4, the cumulative drug release rate was 24.5% within 4 h (Fig. 3a). After NIR irradiation, the cumulative release rate of the drug reached 80.6% within 4 h, which was 3.3 times higher than that of the untreated group. The increase of release rate after NIR irradiation is due to the heat energy produced by PEG-BPEI-rGO, which destroyed the π–π conjugation between DOX and carrier. In vitro drug release experiments show that NIR irradiation can cause the rapid release of DOX, and the drug delivery system has the effect of NIR controlled release.

As shown in Fig. 3b, zeta potential measurements showed that GO is negatively charged (− 35.4 mV). After connecting BPEI, the modified BPEI-GO exhibits a positive charge (39.9 mV) due to the positive charge of amino bands on BPEI. After further modification of PEG, the positive charge of PEG-BPEI-rGO decreases due to the negative charge of PEG, and the surface potential of PEG-BPEI-rGO is 12.1 mV. The change of surface potential of nanocarriers further illustrates the successful combination of BPEI and PEG with GO. We investigated the fluorescence signal of DOX after loading on the PEG-BPEI-rGO. As shown in Fig. 3c, the fluorescence of DOX quenched after loading on the surface of PEG-BPEI-rGO due to the photoinduced electron-transfer effect. However, the fluorescence of DOX recovered by addition of ethanol. The ethanol could destruct the noncovalent interaction between DOX and PEG-BPEIrGO, leading to the fluorescence recovery of DOX.

### Preparation and characterization of MAs-DOX/PEG-BPEI-rGO

**Fig. 4:**
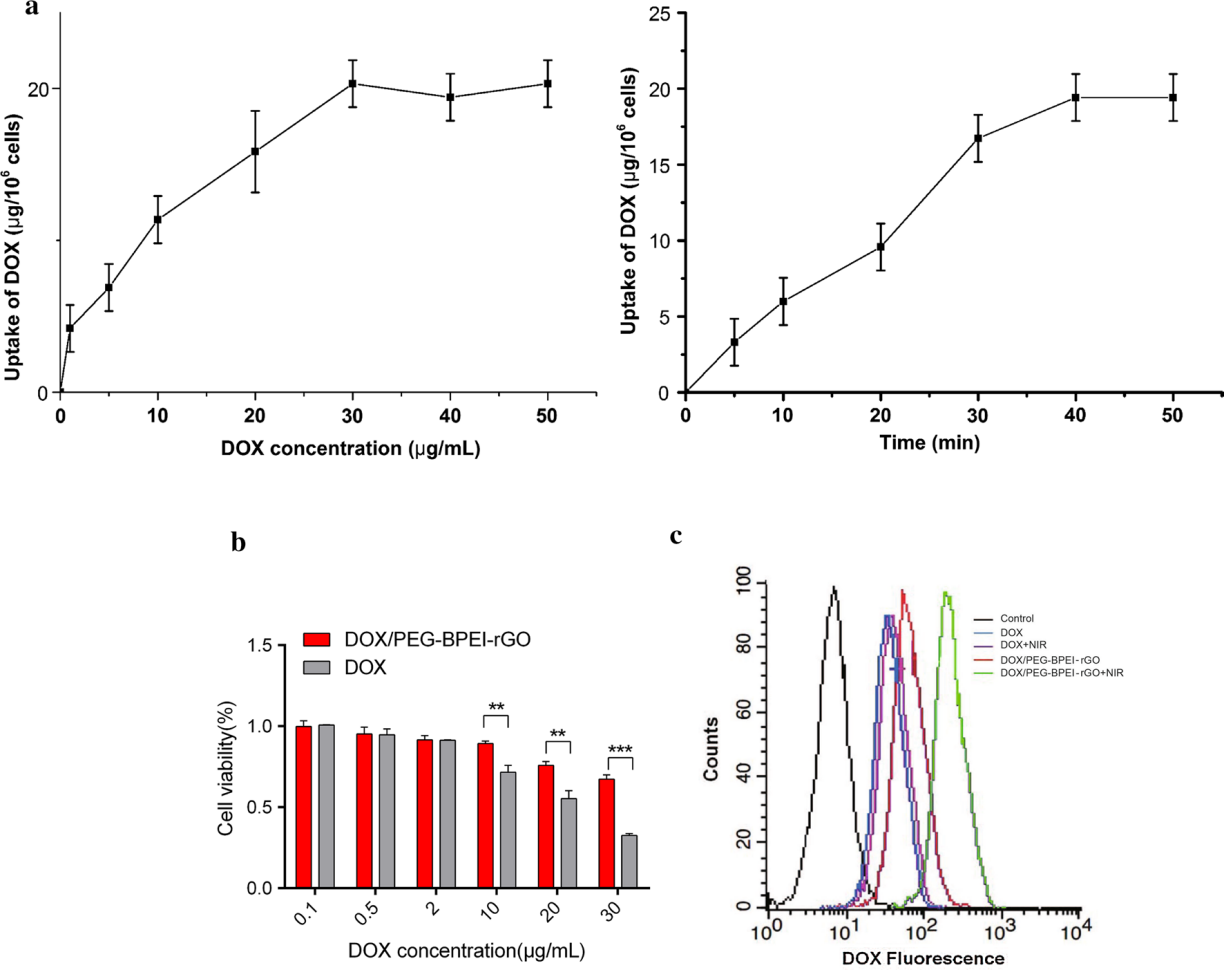
**a** Effects of drug concentration and incubation time on drug loading of macrophages. Data are expressed as mean ± SD (n = 3). **b** Cytotoxicity of DOX and DOX/PEG-BPEI-rGO towards RAW264.7 cells for 12 h. Data are expressed as mean ± SD (n = 3). **p < 0.01, ***p < 0.001. **c** Flow cytometry profiles of DOX fluorescence from DOX/PEG-BPEI-rGO and free DOX in RAW264.7 cells under different NIR irradiation conditions

We incubated macrophages with different concentrations of DOX/PEG-BPEI-rGO for 50 min to investigate the effect of concentration on drug loading of macrophages. The results showed that when DOX concentration in the carrier was under 30 μg/mL, the content of DOX loaded by macrophages increased with the increase of DOX/PEG-BPEI-rGO concentration. When the concentration exceeded 30 μg/mL, the drug loading of macrophages did not change significantly with the increase of concentration. We further investigated the effect of incubation time on drug loading ability of macrophages. With the increase of incubation time, drug loading of macrophages increased continuously. When incubation time exceeded 40 min, drug loading of cells did not increase significantly. Based on the above data (Fig. 4a), we finally determined that the drug loading method of macrophages was: incubating with the PBS solution of DOX/PEG-BPEI-rGO (DOX 30 μg/mL) for 40 min. The constructed MAs-DOX/PEG-BPEI-rGO had a loading capacity of 20 μg DOX/10^6^ cells.

Knowing that the essential requirement of a biomimetic drug delivery system is that the drug-loaded cells can maintain their activity after drug loading we detected cell viability of the macrophages by CCK-8 assay. When DOX was bound to the surface of PEG-BPEI-rGO and little free DOX was released into the cytoplasm before receiving NIR irradiation, DOX/PEG-BPEI-rGO exhibited little effect on the viability of the macrophages at all designated concentrations. Compared with free DOX, DOX/PEG-BPEI-rGO produced a much lower cytotoxicity at high concentrations (Fig. 4b), which provided the basic condition for the macrophages to be loaded with DOX/PEG-BPEI-rGO.

Flow cytometry was used to measure the fluorescence intensity of the whole cell, knowing that it represents the amount of DOX ingested. As illustrated in Fig. 4c, DOX/PEG-BPEI-rGO showed a higher uptake rate than DOX at the same concentration. Due to the electron transfer effect, the fluorescence of DOX was partially quenched in DOX/PEG-BPEI-rGO initially. After receiving the NIR irradiation for 5 min, the DOX signal of the DOX/PEG-BPEI-rGO treated groups shifted to the right, showing a great number of DOX was detached from PEG-BPEI-rGO and recovered fluorescence. However, the macrophages treated with free DOX exhibited little changes in fluorescence signal after receiving NIR treatment.

### NIR-triggered DOX release

**Fig. 5:**
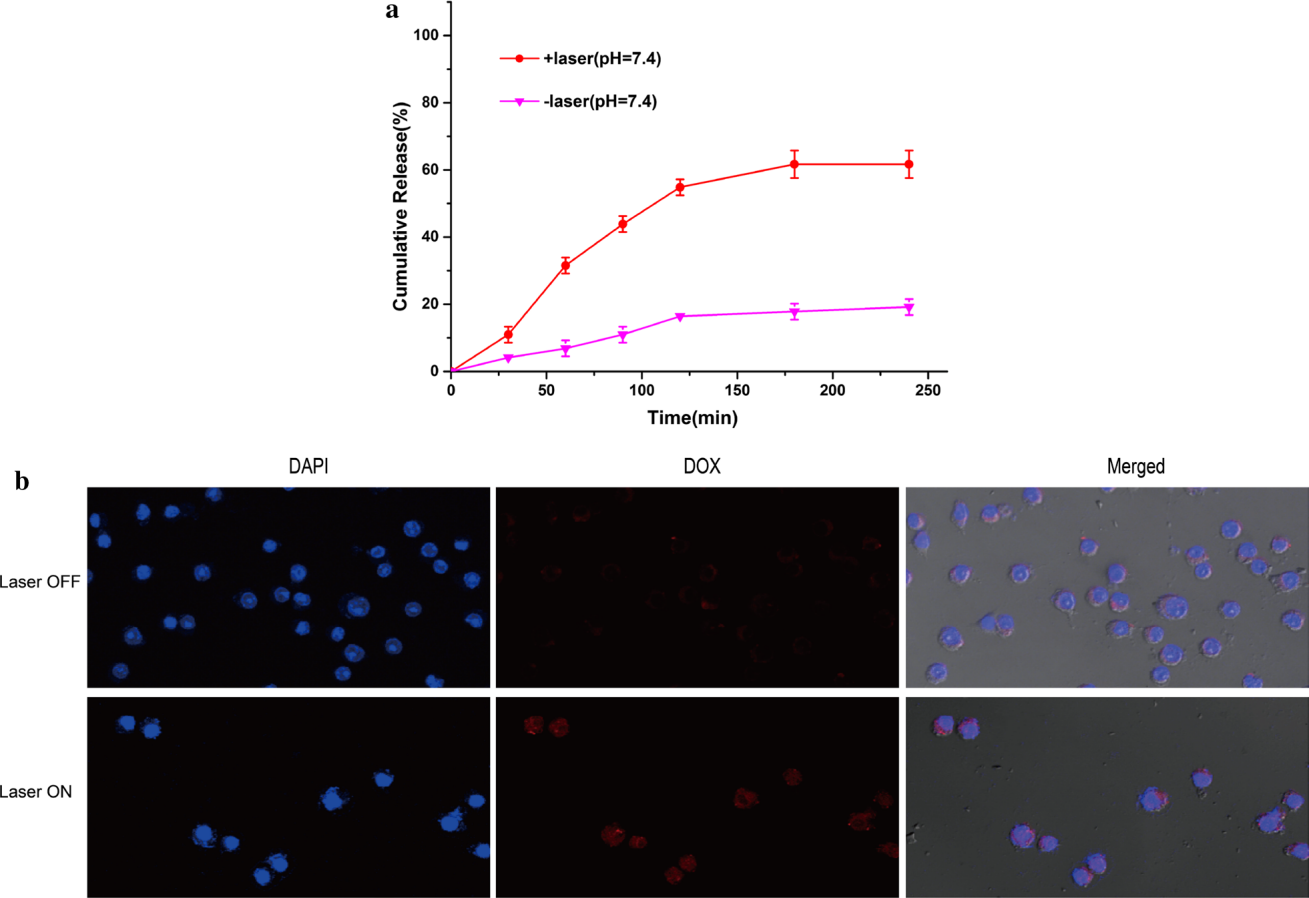
**a** Drug release curves of DOX from MAs-DOX/PEG-BPEI-rGO with or without NIR irradiation. **b** Confocal microscopic images of RAW264.7 cells incubated with DOX/PEG-BPEI-rGO under different NIR irradiation conditions. Nuclei were stained with DAPI

The release ability of DOX from the macrophages under different conditions was further investigated. Under physiological conditions, little DOX was released from the macrophages, indicating that MAs-DOX/PEG-BPEI-rGO could maintain stability during the chemotactic process. The effect of NIR irradiation on DOX release was also investigated. Figure 5a showed that a rapid release of DOX was found after receiving the irradiation (1 W/cm^2^) over 5 min. Under the conditions of pH 7.4, the cumulative release of DOX reached 61.7% within 3 h, which was much higher than that without irradiation. The enhanced release may be due to the disruption of the binding energy between DOX and PEG-BPEI-rGO by NIR-generated heat [21]. These results indicated that MAs-DOX/PEG-BPEI-rGO could maintain stability before reaching the tumor site, and release the encapsulated DOX rapidly upon receiving irradiation. Therefore, MAs-DOX/PEG-BPEI-rGO could be used as an NIR-controlled drug release system.

DOX loaded in the biomimetic drug delivery system was released sequentially: the photothermal heat generated by NIR irradiation caused DOX to release from the surface of PEG-BPEI-rGO, and then the dissociated DOX was excreted out of the macrophages. We further used the CLSM to investigate the intracellular DOX release behavior in the macrophages by incubating the macrophages with DOX/PEG-BPEI-rGO for 40 min and receiving NIR laser for 5 min. Due to the photoinduced electron transfer effect, the fluorescence signal of DOX was very weak before receiving NIR irradiation, and the red DOX signal was only found in the cytosol, indicating that little DOX was released from PEG-BPEI-rGO (Fig. 5b). Following 5 min of NIR irradiation, strong red fluorescence signal was observed in both the nucleus and cytosol. These results indicated that DOX was dissociated from PEG-BPEI-rGO surface into the cytoplasm, and diffused in the nucleus after NIR irradiation. These findings showed that the release of DOX in MAs-DOX/PEG-BPEI-rGO could be intelligently controlled by NIR irradiation.

### The chemotaxis of RAW264.7 cells in vitro

**Fig. 6:**
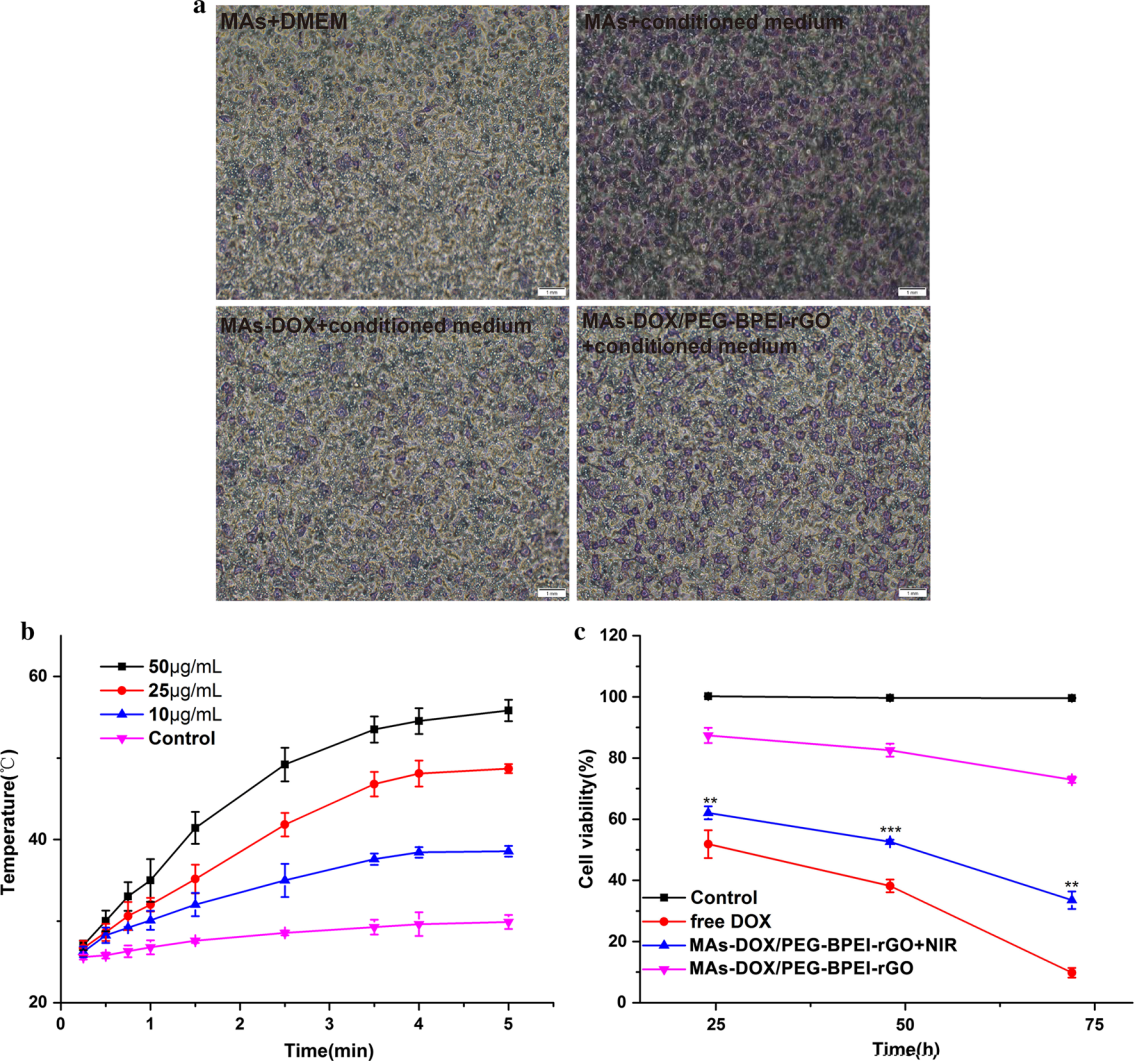
**a** Transwell assay results of RAW264.7 cells towards RM-1 tumor cells in vitro. The migrated RAW264.7 cells were stained with violet crystal. **b** Temperature changes of PBS and aqueous dispersions of MAs-DOX/PEG-BPEI-rGO with different PEG-BPEI-rGO concentrations after 5-min NIR irradiation at a power density of 1 W/cm^2^. **c** The cell viability index of RM-1 cells treated with free DOX, MAs-DOX/PEG-BPEI-rGO centrifugation medium and irradiated MAs-DOX/PEG-BPEI-rGO centrifugation medium. The untreated RM-1 cells were used as control. Results are represented as mean ± SD (n = 3). *p < 0.05, **p < 0.01, NIR treated MAs-DOX/PEG-BPEI-rGO compared with untreated MAs-DOX/PEG-BPEI-rGO (two-tailed Student’s t-test)

Tumor cells such as RM-1 used in this study could secrete large amounts of chemokines and produce a chemical gradient [22]. Then RAW264.7 cells would directly move along the chemical gradient to the tumor site [23]. In this experiment, we used the transwell membrane system to stimulate the chemical gradient of chemokines [24]. The effects of tumor chemokines, chemotherapeutic drugs and drug loaded particles on cell chemotaxic ability were evaluated. As shown in Fig. 6a, when the lower chamber was filled with blank culture medium, few macrophages would enter the lower compartment through the transwell membrane, due to the absence of tumor chemokines. However, when the lower chamber was replaced with the culture medium containing RM-1 cells, the number of macrophages passing through the transwell membrane was enhanced. These results indicated that the macrophages had obvious tumor tropism in vitro cell experiments, which provide a theoretical basis for using macrophages as cellular vehicles towards the tumor site. After being loaded with DOX/PEG-BPEI-rGO, the number of the macrophages moving toward the lower chamber filled with conditioned media of RM-1 cells decreased a little. However, when the macrophages were loaded with DOX directly, the number of the macrophages passing through the transwell membrane decreased, indicating that DOX had a more serious effect on the tumor homing capacity of RAW264.7 cells than DOX/PEG-BPEI-rGO.

### Measurement of the NIR photothermal effect in vitro

PEG-BPEI-rGO exhibited stronger absorption than GO at the 808 nm wavelength, suggesting that PEG-BPEI-rGO may have strong light-to-heat conversion efficiency. This result inspired us to further investigate the photothermal effect of PEG-BPEI-rGO, knowing that it would have a huge impact on photothermal therapy and drug release. We investigated the photothermal conversion efficiency of MAs-DOX/PEG-BPEI-rGO with different PEG-BPEI-rGO concentrations, using an 808 nm laser (1 W/cm^2^). As shown in Fig. 6b, the MAs-DOX/PEG-BPEI-rGO dispersion (PEG-BPEI-rGO 50 μg/mL) could be heated to 55.8 °C in 5 min after receiving NIR irradiation. As a blank control, the temperature of PBS only increased by 4.9 °C after receiving irradiation at the same intensity. These results showed that PEG-BPEI-rGO loaded in the macrophages could efficiently and rapidly convert the light energy into heat. Moreover, when the concentration of PEG-BPEI-rGO was raised from 10 to 50 μg/mL, the temperature of the dispersion could increase by 13.6, 23.7, and 30.8 °C with the same light intensity and exposure time. This result indicated that the dispersion temperature increased as the concentration of PEG-BPEI-rGO increased. The excellent photothermal conversion ability of PEG-BPEI-rGO in vitro encouraged us to carry out the further investigation in vivo.

### Cytotoxicity of DOX released from RAW264.7 cells against RM-1 cells

The cytotoxicity of DOX released from MAs-DOX/PEG-BPEI-rGO against RM-1 cells was investigated. As shown in Fig. 6c, MAs-DOX/PEG-BPEI-rGO treated with NIR irradiation could produce a significant anti-proliferation effect, indicating that DOX released from the macrophages still retained its bioactivity. As DOX was not completely released from the macrophages after irradiation, the free DOX group exhibited stronger cytotoxicity than the NIR treated MAs-DOX/PEG-BPEI-rGO group under the same concentration. However, the untreated MAs-DOX/PEG-BPEI-rGO group exhibited rather low cytotoxicity, indicating that little DOX was excluded out of the macrophages before receiving the NIR irradiation. These results indicated that MAs-DOX/PEG-BPEI-rGO could effectively release DOX after NIR treatment, and showed obvious anti-tumor effect.

### The tumor-targeting ability of macrophages in vivo

**Fig. 7:**
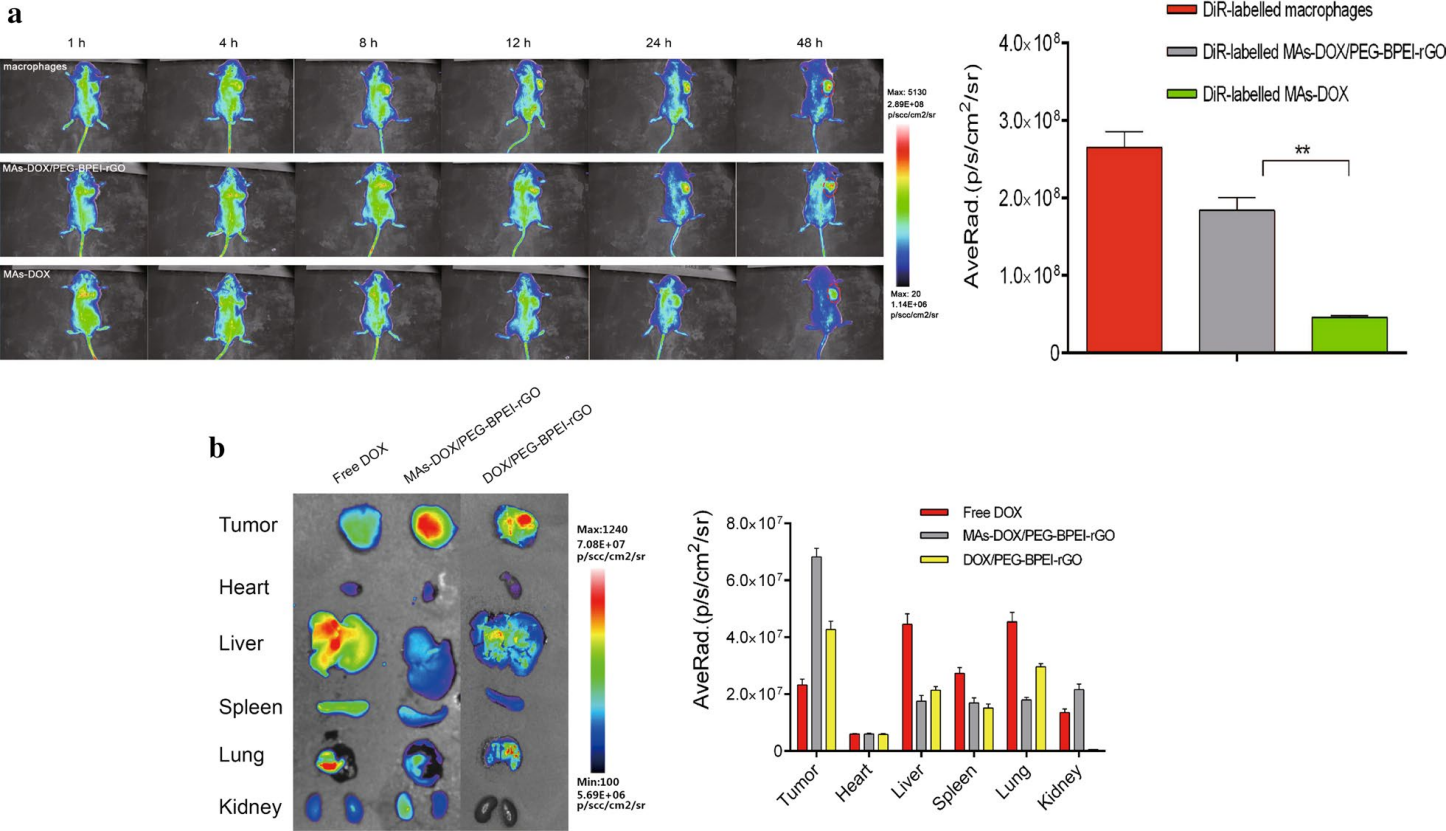
**a** In vivo fluorescence imaging of tumor-bearing mice after IV injection of DiR-labeled RAM264.7 cells, DiR-labeled MAs-DOX/PEG-BPEI-rGO and DiR-labeled MAs-DOX at different time points. Fluorescence intensities of the tumor sites at 48 h were quantified by the Living Image software. **b** Fluorescence distribution of DOX in tumors and major organs after IV injection of free DOX, MAs-DOX/PEG-BPEI-rGO with NIR treatment and DOX/PEG-BPEI-rGO with NIR treatment. Fluorescence intensities were quantified by Living Image software. Data are expressed as mean ± SD (n = 3), *p < 0.05, **p < 0.01

The chemotaxis of RAW264.7 cells toward RM-1 tumor cells was confirmed by the transwell assay in vitro. To further investigate the tumor homing ability of RAW264.7 cells in vivo, the location of DiR-labeled RAW264.7 cells was monitored in the tumor-bearing mice after IV injection. The live mice were scanned at 1, 4, 8, 12, 24 and 48 h post injection (Fig. 7a). The signal of DiR-labeled macrophages was observed in the tumor site as early as 1 h, and the signal strength increased in the following time. This phenomenon indicated that macrophages also exhibited obvious tumor homing ability in vivo. The effect of DOX and DOX/PEG-BPEI-rGO on the physiological behavior of the macrophages were further investigated, the macrophages loaded with DOX or DOX/PEG-BPEI-rGO were labeled by DiR and injected IV into the mice. After being loaded with DOX/PEG-BPEI-rGO, the signals of the macrophages were identifiable 1-h after injection and remained detectable for 48 h. There was no significant change in macrophage distribution in vivo, indicating that DOX/PEG-BPEI-rGO had little effect on the tumor homing ability of the macrophages. However, the fluorescence intensity at the tumor site was much weaker in the group treated with DOX-loaded macrophages, indicating that DOX generated a greater impact on chemotaxis of the macrophages than DOX/PEG-BPEI-rGO, which is consistent with the result of the transwell assay in vitro.

### DOX accumulation in vivo

The study of dynamic distribution of the macrophages showed that loading DOX/PEG-BPEI-rGO into the macrophages did not affect their tumor homing ability, and the macrophages could be efficiently enriched at the tumor site after IV injection. These results encouraged us to investigate the DOX accumulation in different organs in vivo. The mice in the MAs-DOX/PEG-BPEI-rGO treated group and DOX/PEG-BPEI-rGO treated group received NIR irradiation at the tumor site 24 h after administration. The free DOX treated group served as control. The major organs and tumors were harvested 2 h after the laser treatment and visualized by an IVIS. Due to the low selectivity of DOX distribution in vivo, the fluorescence intensity of tumor in the control group was weak, with only a small amount of DOX enriched in the tumor after injection. The tumors in the group treated with DOX/PEG-BPEI-rGO and NIR irradiation exhibited stronger fluorescence signals than the control group due to the EPR effect. The fluorescence signals of tumors in the group treated with MAs-DOX/PEG-BPEI-rGO and NIR were much stronger than that in the group treated with DOX/PEG-BPEI-rGO and NIR irradiation (Fig. 7b), indicating that the macrophages could efficiently transfer DOX/PEG-BPEI-rGO to the tumor site via their inherent tumor homing ability. We also investigated the DOX accumulation in different organs. DOX accumulation in the spleen of the MAs-DOX/PEG-BPEI-rGO treated group was relatively higher than that of the free DOX treated group and DOX/PEG-BPEI-rGO treated group, mainly due to the inherent migratory capacity of the macrophages to this reservoir. Compared with the group treated with free DOX and the group treated with DOX/PEG-BPEI-rGO, the fluorescence intensities of DOX in other organs were lower in the MAs-DOX/PEG-BPEI-rGO treated group.

### The NIR photothermal effect in vivo

**Fig. 8:**
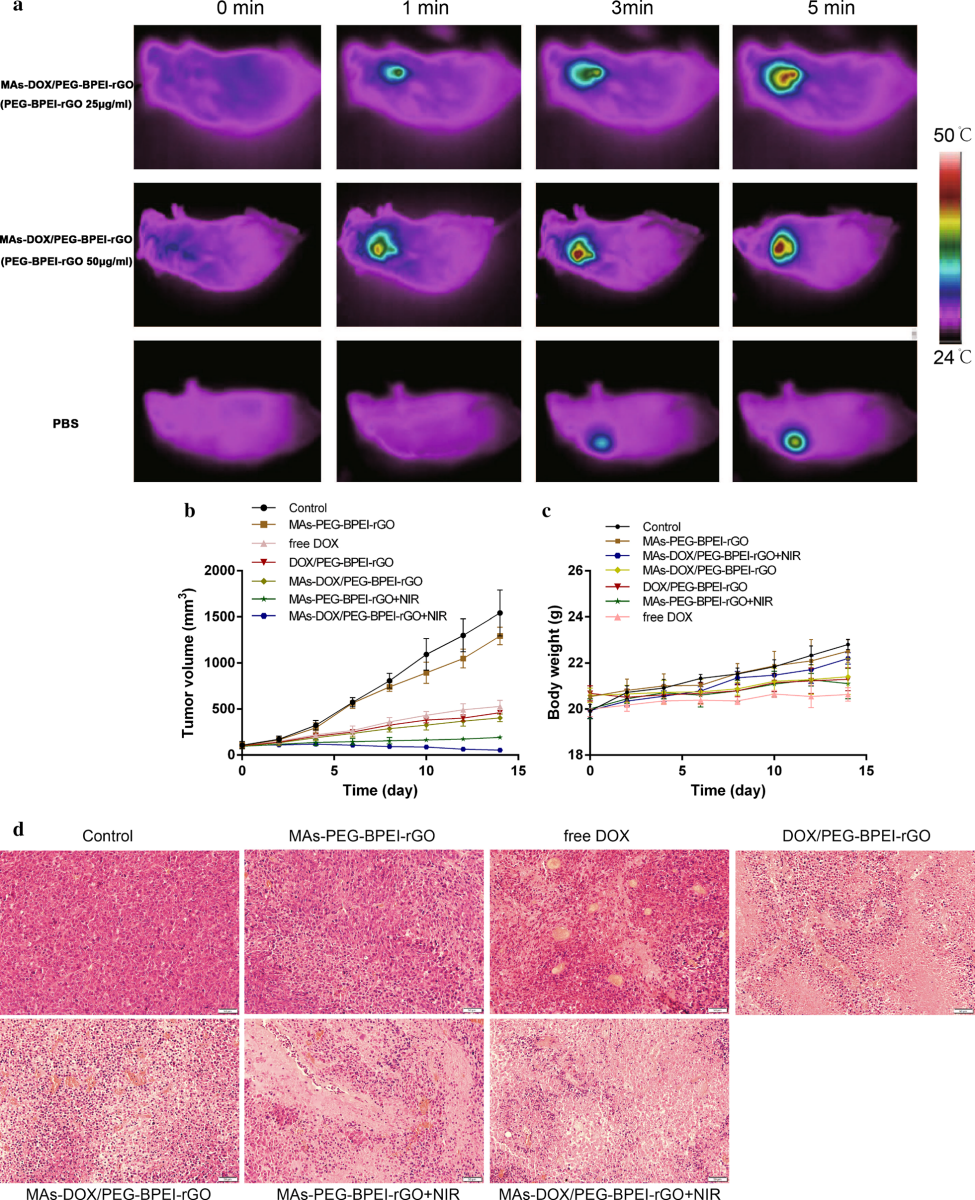
**a** Thermographic images of the tumor-bearing mice treated with PBS or MAs-DOX/PEG-BPEI-rGO 4 h after injection following NIR irradiation for 5 min (1 W/cm^2^). **b** Relative tumor volume in different treating groups (n = 5). **d** Histological photomicrographs of H&E-stained tumor sections from the mice in different groups. **c** Changes of body weight in response to different treatments (n = 5), ***p < 0.01

An IR thermal camera was used to record the tumor temperature change. The mice treated with PBS and NIR irradiation were set as controls. In the PBS group, temperature increased only 4 °C after irradiation at the tumor site. The temperature at the tumor site rose to 41.7 °C and 46.3 °C in MAs-DOX/PEG-BPEI-rGO (PEG-BPEI-rGO 25 μg/mL) and MAs-DOX/PEG-BPEI-rGO (PEG-BPEI-rGO 50 μg/mL) groups after receiving 5 min irradiation (Fig. 8a), respectively. PEG-BPEI-rGO accumulated at the tumor site exerted an excellent photo-thermal conversion effect, which effectively converted the external light energy into heat.

### Antitumor efficacy in vivo

The antitumor efficacy was also investigated on the tumor-bearing mice. The tumor-bearing mice were divided into seven experimental groups, using PBS as the control. Figure 8a showed that the tumor temperature increased to 46.3 °C after treating with MAs-DOX/PEG-BPEI-rGO (PEG-BPEI-rGO 50 μg/mL) and NIR laser. Due to the good photothermal conversion efficiency of PEG-BPEI-rGO, the growth of tumors was significantly suppressed in the group administrated with MAs-PEG-BPEI-rGO and irradiation. However, tumors in the group receiving only MAs-PEG-BPEI-rGO exhibited a growth rate similar to that in the control group. The group treated with MAs-DOX/PEG-BPEI-rGO and irradiation showed a better tumor inhibition effect than the group treated with MAs-PEG-BPEI-rGO and irradiation, which confirmed the in vivo synergistic effect of chemo-photothermal therapy (Fig. 8b). Due to the tumor targeting of macrophages, the tumor size in the group treated with MAs-DOX/PEG-BPEI-rGO was smaller than that treated with DOX/PEG-BPEI-rGO. The tumor size in the group treated with MAs-DOX/PEG-BPEI-rGO and NIR laser was significantly smaller than that treated with MAs-DOX/PEG-BPEI-rGO alone, revealing the importance of NIR-controlled DOX release from the cellular carrier. As shown in Fig. 8d, the tumor tissue sections in the group receiving MAs-DOX/PEG-BPEI-rGO and irradiation exhibited more severe cancer necrosis and a lower nuclear-to-cytoplasmic ratio than the other groups. Meanwhile, no significant weight loss was noticed during the experiment (Fig. 8c). These empirical findings demonstrated that heat generated by MAs-DOX/PEG-BPEI-rGO upon irradiation enhanced the chemotherapy effect of DOX, showing an obvious synergistic chemo-photothermal effect. The excellent in vivo therapeutic effect and biocompatibility of the new biomimetic drug delivery system inspired us to carry out further study in different tumor models.

## Discussion

At the same DOX concentration, macrophages can absorb more DOX/PEG-BPEI-rGO, indicating that the graphene carrier can effectively help macrophages to load more drugs. In addition to the high drug loading caused by the large surface area of PEG-BPEI-rGO, the large size of PEG-BPEI-rGO also promotes the loading efficiency of macrophages. Since macrophages are phagocytic cells, matrix metalloproteinases present on the surface of macrophages can easily recognize larger particles, thereby activating phagocytosis of macrophages [25, 26].

As a tumor-targeting vector, macrophages should maintain biological activity after drug loading. As shown in Fig. 4b, cell viability in DOX/PEG-BPEI-rGO group was higher than that in DOX group, indicating that the PEG-BPEI-rGO carrier could protect the macrophages against DOX toxicity. The low cytotoxic effect of DOX/PEG-BPEI-rGO might be attributed to the controlled DOX release from PEG-BPEI-rGO. As only a small amount of DOX was released from the graphene carrier into the nucleus before receiving irradiation, DOX/PEG-BPEI-rGO exhibited little effect on the physiological activity of the macrophages. The transwell assay results indicate that the cancer-targeting ability of the macrophages was still promising after loading DOX/PEG-BPEI-rGO, which provided a basis for in vivo tumor targeting investigation.

As MAs-DOX/PEG-BPEI-rGO could maintain stability before getting NIR irradiation, only a small amount of DOX was released into the circulatory system, and most DOX was delivered to the tumor site and released upon NIR irradiation. NIR-controlled release at the tumor site might can enhance the cancer cell killing effect and reduce the toxic effect on other normal organs and tissues. Fluorescent images of DOX in the tumor and different organs after IV administration showed that the macrophages could effectively transport DOX/PEG-BPEI-rGO to the tumor site (Fig. 7b), reducing the toxicity to other organs. The non-specific distribution of DOX is the most important factor for its toxicity to healthy tissues, thus limiting its clinical use of high doses which is essential in killing tumor cells [27]. The biomimetic drug delivery system described in this study can effectively solve this problem by enhancing the tumor targeting ability of DOX/PEG-BPEI-rGO.

Graphene used in this biomimetic drug delivery system is a photothermal conversion material with strong absorption in the near-infrared range. However, further research showed that graphene may accumulate in the body due to slow degradation, which may limit its clinical transformation and application. In future study, we will try to combine macrophages with some biodegradable photothermal materials such as black phosphorus [28].

As RAW264.7 cells are a kind of tumor origin cells, their fate after injection needs particular attention. It was reported that RAW264.7 activated by IL-4 and LPS promoted the growth of lung cancer in a mouse model [29]. In our study, RAW264.7 cells loaded with DOX/PEG-BPEI-rGO would be destructed and metabolized after receiving NIR irradiation at the tumor site, due to the heat generated by PEG-BPEI-rGO and the released DOX. In future clinical trials, we can collect macrophages from the patients themselves, culture them in vitro, load them with therapeutic agents, and finally re-infuse them back into the patients. Of course, more animal experiments are required to confirm the safety before it can be applied to clinical practice.

## Conclusions

We have developed a macrophages-based biomimetic drug delivery system by using the physiological characteristics of macrophages to transport DOX-loaded PEG-BPEI-rGO to the tumor site and deliver DOX to tumor cells in an on-demand manner upon NIR irradiation. MAs-DOX/PEG-BPEI-rGO was prepared by loading DOX/PEG-BPEI-rGO into macrophages, which would retain their biological activity to ensure their active targeting to tumor cells. Upon MAs-DOX/PEG-BPEI-rGO receiving NIR irradiation after arriving at the tumor site, DOX would be dissociated from the surface of the functionalized PEG-BPEI-rGO by the heat produced by photothermal effect. At the same time, the integrity of the macrophages was disrupted, resulting in the rapid release of encapsulated DOX. Using the photothermal effect produced by PEG-BPEI-rGO and DOX released from the macrophages upon NIR irradiation, MAs-DOX/PEG-BPEI-rGO exhibited a significant inhibitory effect on tumor growth.

## Methods

### Materials

Materials used in this study were graphene oxide (GO) solution (Xianfeng Biological Technology Co., Ltd., Jiangsu, China); 1-ethyl-3-[3-(dimethylamino)propyl] carbodi-imide hydrochloride (EDC), *N*-hydroxysuccinimide (NHS), triethylamine (TEA) and 80% hydrazine monohydrate (Yuanye Biological technology Co., Ltd., Shanghai, China); polyethylene glycol monomethyl ether (methoxy PEG, Mw: 3 kDa) and branched polyethylenimine (BPEI, Mw: 1.8 kDa) (Sigma-Aldrich, St. Louis, MO); Dulbecco’s modified Eagle’s medium (DMEM), trypsin, fetal bovine serum (FBS), violet crystal and Cell Counting kit-8 (CCK-8) (KeyGEN Bio TECH, Jiangsu, China); and doxorubicin (DOX, 99%) (Aladdin Co., Ltd., Shanghai, China). The other chemicals and reagents were of analytical grade.

Male Balb/c mice aged 6 weeks were purchased from Second Military Medical University Animal Care Center (Shanghai, China) and kept under SPF condition with free access to standard food and water. All the experiments were carried out according to the principles of care and use of Laboratory animals set by the Laboratory Animal Center of The Second Military Medical University of China.

### Synthesis of PEG-BPEI-rGO

In the present study, we constructed a functionalized reduced graphene oxide (rGO) as a platform for integrated chemo-photothermal therapy. We synthesized the functionalized rGO according to the method reported by Kim et al. In brief, EDC (54 mg) and NHS (50 mg) were added to the GO solution (0.5 mg/mL) to active the carboxylic acid group of GO. Then the BPEI solution (400 mg) containing TEA (100 μL) was added to the GO solution and the mixture was stirred at room temperature for 24 h. The resulting BPEI-GO solution was dialyzed in a dialysis bag (MWCO: 3.5 K) for 24 h to remove the unreacted material [18]. The reducing process was conducted by adding 0.05 v/v hydrazine monohydrate to the BPEI-GO solution followed by heating to 80 °C for 15 min. Activated PEG (3 K, 5 mmol) solution was added to BPEI-rGO in deionized water to increase the hydrophilicity BPEI-rGO. After stirring for 20 h, the resulting product was dialyzed in a dialysis bag (MWCO: 5 K) for 24 h.

### Characterization of PEG-BPEI-rGO

The morphology and size of GO and PEG-BPEI-rGO were characterized by atomic force microscopy (AFM). Droplets of GO and PEG-BPEI-rGO dispersion (0.05 mg/mL) were added on the freshly prepared Mica plate and dried at room temperature. Fourier transform infrared spectroscopy (FTIR) analyses were carried out to confirm the successful modification of GO. Raman Spectroscope and UV/vis spectrophotometry were used to verify the successful reduction process.

### Drug loading and release of PEG-BPEI-rGO

To load DOX onto the surface of PEG-BPEI-rGO, 4 mL DOX DMSO solution (0.5 mg/mL) was added to 4 mL PEG-BPEI-rGO dispersion (0.25 mg/mL) and stirred for 12 h at room temperature. To remove the unbound DOX, the mixture was dialyzed against distilled water for 12 h. DOX/PEG-BPEI-rGO was washed twice by filtration (MWCO: 3000 Da) and centrifuged at 6000 rpm for 15 min. The absorbance of DOX/PEG-BPEI-rGO aqueous solution at 520 nm was measured by UV spectrophotometry, and the absorbance of PEG-BPEI-rGO was subtracted. The absorbance value was brought into the standard curve of DOX. The drug loading capacity (DLC) of PEG-BPEI-rGO was calculated according to the formula.

$$ {\text{DLC }} = {\text{W}}_{\text{DOX}} /{\text{W}}_{\text{PEG - BPEI - rGO}} \times 100\% $$

where W_DOX_ represent the weight of DOX loaded on PEG-BPEI-rGO, W_PEG-BPEI-rGO_ represent the total weight of PEG-BPEI-rGO.

We investigated the release of DOX from DOX/PEG-BPEI-rGO in vitro. 2 mL DOX/PEG-BPEI-rGO PBS solution (containing DOX 200 μg) was transferred to the dialysis bag (3000 Da) against 40 mL PBS (pH 7.4). Laser impact on DOX release was carried out by 808 nm laser (1 W/cm^2^) over 5 min. 1 mL sample was collected at predetermined time intervals (0–3 h). The amount of DOX released into the medium was evaluated by measuring the UV absorbance at 520 nm.

### Preparation of MAs-DOX/PEG-BPEI-rGO

DOX/PEG-BPEI-rGO was loaded into the macrophages to prepare the DOX/PEG-BPEI-rGO-loaded macrophages delivery system (MAs-DOX/PEG-BPEI-rGO). Briefly, macrophages (1 × 10^5^ cells) were suspended in 2 mL DOX/PEG-BPEI-rGO PBS dispersion with different concentration (DOX concentration was 1, 5, 10, 20, 30, 40, 50 μg/mL respectively) at 37 °C for 50 min. MAs-DOX/DOX/PEG-BPEI-rGO were collected by centrifugation for 5 min at the speed of 3000 rpm. The drug-loaded RAW264.7 cells were re-suspended in PBS. To quantify the DOX content in MAs-DOX/PEG-BPEI-rGO, ethanol of the same volume was added to the supernatant after centrifugation to dissociate DOX from the surface of PEG-BPEI-rGO. Ultrafiltration (MWCO: 3000 Da) was used to filter DOX. The absorbance of filtrate at 520 nm was measured by UV spectrophotometer, and the standard curve was introduced to calculate the DOX content which was not absorbed by RAW264.7 cells. The DL capacity of the macrophages was calculated according to the formula.

$$ {\text{DL}} = \frac{{{\text{M}}_{\text{total}} - {\text{M}}_{\text{left}} }}{{{\text{N}}_{\text{cell}} }} \times 10^{6} \times 100 $$

where ${\text{M}}_{\text{total}}$, ${\text{M}}_{\text{left}}$ and ${\text{N}}_{\text{cell}}$ represent the total amount of DOX, the amount of DOX remaining after co-culture, and the total amount of cells, respectively [11].

Macrophages (1 × 10^5^ cells) were dispersed in 2 mL DOX/PEG-BPEI-rGO PBS solution (DOX concentration was 30 μg/mL) and incubated for different times (5 min, 10 min, 20 min, 30 min, 40 min and 50 min respectively) at 37 °C. MAs-DOX/DOX/PEG-BPEI-rGO were collected by centrifugation for 5 min at the speed of 3000 rpm. The drug loading of cells was calculated according to the above methods, and the effects of incubation time on drug loading were analyzed.

### Cytotoxicity of DOX/PEG-BPEI-rGO towards macrophages

The cytotoxicity of DOX/PEG-BPEI-rGO against the macrophages was measured using the CCK-8 assay. The blank macrophages (8 × 10^3^ cells/well) were seeded into the 96-well plates. After 24-h incubation at 37 °C, the culture medium was removed. DOX with different concentration (0.1 μg/mL, 0.5 μg/mL, 2 μg/mL, 10 μg/mL, 20 μg/mL, 30 μg/mL) and DOX/PEG-BPEI-rGO containing the same DOX concentration were added into the wells. After 40-min co-incubation at 37 °C, PBS was used to wash the cell. After incubation for another 12 h, 10 μL CCK-8 medium was added to each well and incubated for 1 h. A microplate reader was used to measure the absorbance at 450 nm. The results were calculated as the mean ± SD (n = 3).

### Flow cytometric analysis

The macrophages were seeded in 12-well culture plates (3 × 10^5^ cells/well, n = 3), and incubated at 37 °C for 24 h in a humidified 5% CO_2_ atmosphere. After co-incubation with free DOX and DOX/PEG-BPEI-rGO containing the same amount of DOX for 40 min, the cells were washed twice. Then, the macrophages were irradiated by the 808 nm laser (1 W/cm^2^) over 5 min. The harvested cells were suspended in DPBS solution with 2% FBS and analyzed with a flow cytometer (FACS Calibur, BD Bioscience, UK).

### NIR-triggered DOX release

We investigated the release of DOX in vitro with a dialysis plastic tube (MW cut-off: 3000 Da). 2 mL MAs-DOX/PEG-BPEI-rGO dispersion (1 × 10^6^ cells/mL) was loaded in the tube against 25 mL PBS (pH 7.4). Laser impact on DOX release was carried out by 808 nm laser (1 W/cm^2^) over 5 min. 1 mL sample was collected at predetermined time intervals (0–3 h). The amount of DOX released into the medium was evaluated by measuring the UV absorbance at 520 nm. After UV spectrophotometry, the sample was returned to the original dispersion.

We further investigated intracellular DOX release after receiving NIR irradiation [30]. In brief, the macrophages were seeded at a density of 1 × 10^4^ cells/well in confocal dishes. Cells were incubated with DOX/PEG-BPEI-rGO for 40 min at 37 °C. Then, MAs-DOX/PEG-BPEI-rGO were irradiated by 808 nm laser (1 W/cm^2^) over 5 min. After two washes with cold PBS and fixation in 4% paraformaldehyde (V/V), the nuclei of the macrophages were stained with DAPI. The cells on coverslips were sealed with mounting medium and observed under a confocal laser scanning microscope (CLSM) (Nikon, Japan).

### Chemotaxis of RAW264.7 cells towards RM-1 tumor cells in vitro

Transwell assay was used to evaluate chemotaxis of RAW264.7 cells towards RM-1 tumor cells in vitro. The lower chamber of the 24-well plate was filled with 600 μL DMEM media or DMEM containing tumor cell. 5 × 10^4^ RAW264.7 cells with or without drug loading in 100 μL FBS-free DMEM media were added to the supernatant wells. After 24-h incubation, the transwell was washed with PBS and fixed in 4% paraformaldehyde solution for 20 min. The migrated RAW264.7 cells were stained with violet crystal and detected under the microscope.

### Cytotoxicity of DOX released from RAW264.7 cells against RM-1 cells in vitro

RM-1 tumor cells were seeded in 96-well plates at a density of 10^4^ per well and incubated at 37 °C in 5% CO_2_ for 24 h. The MAs-DOX/PEG-BPEI-rGO dispersion (1 × 10^5^ cells/mL, equivalent to 2 μg/mL DOX) received NIR irradiation (1 W/cm^2^) for 5 min. 2 h after NIR treatment, both the treated and untreated MAs-DOX/PEG-BPEI-rGO was centrifuged for 6 min at 5000 rpm. The collected supernatant and free DOX (2 μg/mL) were added to the tumor cells and incubated for 24, 48 and 72 h, using the untreated RM-1 cells as control. After that, 10 μL CCK-8 medium was added. After co-incubation for 4 h, a microplate reader was used to measure the absorbance at 450 nm.

### Macrophages biodistribution in vivo

A tumor bearing mouse model was established by subcutaneous inoculation of RM-1 cells (5 million) into the right flank of male BALB/c mice. To access the biodistribution and tumor targeting abilities of the macrophages in vivo, the cell membranes of macrophages were first stained with DiR. RAW264.7 cells were resuspended in PBS with a cell density of 1 × 10^6^/mL. Cell dispersions were added to DiR solution (10 μg/mL) at a volume ratio of 1:1. The cells were cultured for 30 min under dark conditions at 37 °C. The stained cells were centrifuged for 30 min at a speed of 1500 rpm. The supernatant was discarded and washed twice with PBS. Then the cells were resuspended with PBS. The staining method of macrophages carrying DOX/PEG-BPEI-rGO or DOX was the same as above. The labeled macrophages (5 × 10^6^ cells/mouse) were intravenously given to the mice. DiR signals were captured at predetermined time intervals by in vivo imaging system (IVIS, Perkin Elmer). Fluorescence intensities of the tumor sites at 48 h were quantified by the Living Image software.

### In vivo DOX accumulation

MAs-DOX/PEG-BPEI-rGO, DOX/PEG-BPEI-rGO and free DOX were injected IV into the mice (tumor volume 70 mm^3^). NIR irradiation (1 W/cm^2^) was conducted on the MAs-DOX/PEG-BPEI-rGO treated group and DOX/PEG-BPEI-rGO treated group 24-h after injection. 2-h after NIR treatment, the mice were sacrificed. Tumors and the major organs were harvested for ex vivo imaging using the IVIS imaging system (Perkin Elmer) [31]. The excitation wavelength was 480 nm, and the images were taken through a 580 nm-emission filter. Fluorescence intensities of different organs and tumors were quantified by the Living Image software.

### Measurement of the NIR photothermal effect

0.5 mL aqueous dispersion of MAs-DOX/PEG-BPEI-rGO with different PEG-BPEI-rGO concentrations (10, 25 and 50 μg/mL) was added to the tube and exposed to 808 nm NIR laser (1 W/cm^2^) for 5 min. A digital infrared thermal camera was applied to measure the thermal imaging and temperature changes at predetermined intervals. PBS was used as control.

The photothermal effect was also investigated on the RM-1 tumor bearing mice. 300 μL MAs-DOX/PEG-BPEI-rGO dispersion with the PEG-BPEI-rGO concentration of 50 μg/mL and 25 μg/mL was injected IV into the mice, using PBS as control. After 24-h IV administration, the tumor site was irradiated with 808 nm laser (1 W/cm^2^) for 5 min. Temperature changes of the tumor site were recorded by an IR thermal camera during irradiation [32].

### Anti tumor efficacy

The therapeutic efficacy was investigated on the RM-1 tumor-bearing mice. The tumor bearing mice were equally randomized into seven groups: 1, control group using PBS only; 2, MAs-PEG-BPEI-rGO group; 3, free DOX group; 4, DOX/PEG-BPEI-rGO group; 5, MAs-DOX/PEG-BPEI-rGO group; 6, MAs-PEG-BPEI-rGO + NIR group; 7, MAs-DOX/PEG-BPEI-rGO + NIR group. The dose of DOX injected in each group was at a rate of 2 mg/kg body weight, on alternative days. In the irradiation groups, NIR irradiation (1 W/cm^2^) was performed 24 h after each IV administration for 5 min. The body weight and tumor size were examed every other day. A caliper was used to measure the tumor size. The tumor volume was calculated according to the formula: V = LW^2^/2, where L and W refer to the long and short diameter of the tumor respectively. All mice were sacrificed after the last operation, the tumor tissue and major organs were sectioned and stained with hematoxylin and eosin (H&E).

### Date analysis

The results are presented as mean ± standard deviation (SD). All the experiments were repeated at least three times. Pairs of groups were compared using the *T*-test in SPSS software version 17.0. Differences were considered to be statistically significant when p < 0.05.

## Data Availability

All dates generated and analyzed during this study are included in this paper.

## Abbreviations

AFM: atomic force microscopy
rGO: reduced graphene oxide
NIR: near-infrared laser
BDS: biomimetic delivery system
MAs: macrophages
CLSM: confocal laser scanning microscopy
IV: intravenously
FTIR: Fourier transform infrared spectroscopy
IVIS: in vivo imaging system
